# Genetic diversity at the *Dhn3* locus in Turkish *Hordeum spontaneum* populations with comparative structural analyses

**DOI:** 10.1038/srep20966

**Published:** 2016-02-12

**Authors:** Cüneyt Uçarlı, Liam J. McGuffin, Süleyman Çaputlu, Andres Aravena, Filiz Gürel

**Affiliations:** 1 Department of Molecular Biology and Genetics, Faculty of Science, Istanbul University, Vezneciler 34134, Istanbul, Turkey; 2 School of Biological Sciences, University of Reading, Whiteknights, Reading RG6 6AS, UK

## Abstract

We analysed *Hordeum spontaneum* accessions from 21 different locations to understand the genetic diversity of *HsDhn3* alleles and effects of single base mutations on the intrinsically disordered structure of the resulting polypeptide (*Hs*DHN3). *Hs*DHN3 was found to be YSK_2_-type with a low-frequency 6-aa deletion in the beginning of Exon 1. There is relatively high diversity in the intron region of *HsDhn3* compared to the two exon regions. We have found subtle differences in K segments led to changes in amino acids chemical properties. Predictions for protein interaction profiles suggest the presence of a protein-binding site in *Hs*DHN3 that coincides with the K_1_ segment. Comparison of DHN3 to closely related cereals showed that all of them contain a nuclear localization signal sequence flanking to the K_1_ segment and a novel conserved region located between the S and K_1_ segments [E(D/T)DGMGGR]. We found that *H. vulgare*, *H. spontaneum,* and *Triticum urartu* DHN3s have a greater number of phosphorylation sites for protein kinase C than other cereal species, which may be related to stress adaptation. Our results show that the nature and extent of mutations in the conserved segments of K_1_ and K_2_ are likely to be key factors in protection of cells.

The wild progenitor of barley, *Hordeum vulgare spp. spontaneum* (C. Koch), which is also known as *H. spontaneum* in modern taxonomy, is distributed in the Fertile Crescent and Irano-Turanian region as its primary habitat and the Mediterranean and Central Asia as secondary habitats^1^,^2^. The Southeastern region of Turkey covers the “Anatolian group”, which is one of the diversity centres for *H. spontaneum*^3^. The region – as the north part of the Fertile Crescent– is characterized by hot, dry climate with average temperature of 38 °C in August and monthly average precipitation is 1.2 mm near the Syrian border (Turkish State Meteorological Service 2015). *H. spontaneum* has a high distribution in semiarid regions where the temperature range is extreme; soil types, altitudes and photoperiods are diverse^4^,^5^. Frequently, agricultural fields of barley and wheat in Southeastern Turkey are occupied by *H. spontaneum* during severe drought seasons. These observations together with the data suggest the increased ability to survive and adaption capacity of the *H. spontaneum* populations under harsh environmental conditions. Today, wild relatives and landrace accessions are more important than ever as they are potential resources of genetic variation for drought tolerant crop development.

Dehydrins are group 2 Late Embryogenesis Abundant (LEA) proteins, which were first characterized in cotton seeds as an up-regulated protein group during maturation^6^,^7^. Dehydrins are normally expressed at low levels in cells and are induced by drought, salinity, low temperatures or exogenous application of absisic acid (ABA)^8^,^9^,^10^,^11^. Certain type of dehydrins has also been shown to be expressed constitutively^12^. Six to ten *Dhn* genes have been characterized in *Arabidopsis thaliana*, and eight of them have been found to be linked by duplication, either as tandem repeats or homologous pairs^13^. In barley, 13 dehydrin genes (*Dhn1* to *Dhn13*) have been identified^14^,^15^. Like most of the group 2 LEA proteins, barley dehydrins contain an S-segment of poly-serine residues near to the N-terminus and a lysine-rich 15 amino-acid consensus sequence named K-segment (EKKGIMDKIKEKLPG) near to the carboxyl-terminus^16^. The K-segment occurs in all dehydrins but the number of copies changes from 1 to 11 within a single polypeptide^14^. Dehydrin molecules also contain another conserved sequence tyrosine-rich residues, known as the Y-segment [V/T]D[E/Q]YGNP near to the N-terminus. Sequence analysis of each of the *Dhn* genes demonstrated that their allelic variations have originated from deletion or duplication of Φ domains (named the Φ-segments) in the K-segment and single nucleotide polymorphisms through the entire gene^14^. The Φ-segments are variable motifs rich in polar amino acids (Gly or Ala/Pro) located between or before the K-segment. There are five different subgroups of dehydrins (Y_n_SK_2_, K_n_, K_n_S, SK_n_, and Y_2_K_n_ types) based on the number and position of the conserved segments^17^. *Dehydrin3* (*Dhn*3) and *Dehydrin4* (*Dhn4)* genes are located on barley chromosome 6H as consecutive genes, while other *Dhn* genes are distributed on 3H, 4H, 5H and 6H^14^. Expression patterns of barley *Dhn* genes have been found to be correlated with the known regulatory element compositions in their sequences^14^. Barley *Dhn3* and *Dhn4* genes have been reported as early responsive genes to drought and other stress factors^18^,^19^,^20^,^21^. Both genes have been rapidly up-regulated in drought-tolerant barley (*Hordeum vulgare* L. “Chalbori”) and their transfer and over-expression in *Arabidopsis* conferred tolerance to this plant^18^. There are not many studies on expression profile of Dhn genes of *H. spontaneum* under drought. However, Suprunova *et al.*^22^ has clearly proved *Dhn3* expressional induction in response to drought, similar to barley.

Dehydrins are hydrophilic due to the presence of polar and charged amino acids (Ala, Gly Lys, Asp, Glu, and Ser). They do not have stable three-dimensional structures, thus they are intrinsically disordered proteins (IDPs)^23^. Despite having high flexibility and minimal secondary structure, IDPs are an important class of proteins, functionally related to cell signalling, transcription, and assembly of protein complexes^24^. As cryoprotectants, dehydrins are known to interact with membrane phospholipids, metal ions, and water during stressful conditions^25^,^26^,^27^,^28^. Recent studies also suggest that conserved motifs of dehydrins, such as K-segments, have a role in their intrinsic disordered structure and binding affinity to other molecules and cell membranes^24^,^29^,^30^. IDPs such as dehydrins often gain structural stability when bound to ligands such as membranes, and they may change their oligomeric state when bound to ions^31^. Experimental studies of disordered regions of proteins have been difficult for X-ray diffraction analyses, and often for nuclear magnetic resonance imaging (NMR) due to their flexible nature and so predictive tools are often used in conjunction^32^.

Although there have been many investigations related to structure and functions of dehydrins, the genetic diversity of individual dehydrin proteins in natural populations and its effects on the resultant polypeptide structure are not well known. In the present study, we have selected DHN3 from the dehydrin family for characterization of biochemical features in comparison to other cereal species. Additionally, we identified indels and SNPs within *HsDhn3* alleles to understand the range of mutations in *H. spontaneum* populations of Turkish origin, which represent an important gene pool.

## Materials and Methods

### Plant material

*H. spontaneum* L. accessions collected from 21 locations mostly in Southeastern Turkey, and *Hordeum vulgare* L. cv. Tokak 157/37 (TK157/37) were used in the study (see Supplemantary Table S1). Seeds were germinated and grown in pots filled with soil in a growth chamber (Angelantoni, Ekochl 700) under a short-day photoperiod (8 h light/16 h dark), at a temperature of 25 °C and 50–60% relative humidity. One plant was used from each accession.

### DNA extraction and isolation of *Dhn3* alleles

We extracted genomic DNA from the leaves of wild barley seedlings following the CTAB method^33^. Specific primers (forward: 5′ AGGCAACCAAGATCAACACC 3′ and reverse: 5′ TTCTGCAAGGTAGCCAGACC 3′) were designed to amplify the whole sequence of the *Dhn3* gene based on sequences of *H. vulgare* cv. Dicktoo presented in GenBank database (AF043089.1) using Primer3 software. The specificity of designed primers was confirmed by BLASTN analysis^34^. Genomic DNA amplifications were performed in a 25 μl reaction containing 0.5 U of Dreamtaq DNA polymerase (Thermo Scientific EP0702), 200 μM of each dNTP, 0.4 μM of primer, 2 mM MgCl_2_ and 50 ng genomic DNA. Thermocycling was performed at 95 °C for 5 min, followed by 35 cycles at 95 °C for 1 min, 61 °C for 40 s, 72 °C for 1 min, and a final extension at 72 °C for 10 min. The resulting amplicons were purifed using Wizard^®^ SV Gel and PCR Clean-Up System (Promega, USA) and cloned into pTZ57R/T plasmid vector (Thermo Scientific, USA) according to the manufacturer’s instructions. The cloned *HsDhn3* fragments were sequenced by the dideoxy chain termination method using ABI Prism 310 Genetic Analyzer (Applied Biosystems, USA).

### DNA sequence analysis

The sequencing chromatograms were examined with Chromas Lite 2.1.1 (Technelysium Pty Ltd, Australia) and converted to FASTA format. The vector sequences were removed using web based VecScreen. The nucleotide and predicted amino-acid sequences were compared with sequences in the GenBank and EMBL databases respectively, using BLAST. The intron was identified by aligning to known sequences of *Dhn3* CDS of cv. Dicktoo (AF043089.1). Amino acid sequence alignments of the predicted DHN3 polypeptide and nucleotide sequence alignments were performed using CLUSTALW2^35^ with default parameters. We identified SNPs among the *HsDhn*3 DNA sequences from different genotypes using MEGA 6.06 software^36^.

### Measurement of nucleotide diversity

Nucleotide diversity (π) between the genotypes was calculated as the average of the pairwise nucleotide difference per site between two sequences according to Nei^37^ (1987) using the MEGA 6.06 software. The number of unique haplotypes (h) and haploid diversity (Hd) were measured using DNAsp version 5.10.01^38^.

### Protein sequence analyses

We analysed the physical and chemical properties of DHN3 including molecular weight, theoretical isoelectric point (pI), stability index and hydropaticity index, according to amino acid scale values by Kyte and Doolittle^39^, using the ProtParam tool from Expasy^40^. Putative protein kinase C (PKC) and casein kinase 2 (CK2) phosphorylation sites were predicted using NetPhosK 1.0^41^. The protein sequences of the DHN3 variants were submitted to the IntFOLD server^42^,^43^ to generate alternative 3D models using the latest methodology^44^. Predictions of the intrinsically disordered (natively unstructured) regions in the sequences were generated using DISOclust^45^ and likely disordered and protein binding regions were predicted with DISOPRED3^46^,^47^.

### Public protein sequences of other cereal species

For comparision of biochemical and structural characteristics, we used public sequences of *H. vulgare* (AF043089.1)*, Triticum aestivum* WZY1 (AAL50791*), Triticum urartu* DHN3 (EMS45466.1)*, Aegilops tauschii* DHN3 (EMT24840)*, Brachypoidum distachyon* DHN3-like (XP003574997)*, Zea mays* RAB17 (CAM56274.1)*, Sorghum bicolor* DHN (AAA19693), and *Oryza sativa* (NP001067843) downloaded from NCBI.

## Results

### Sequence Diversity

**Figure 1:**
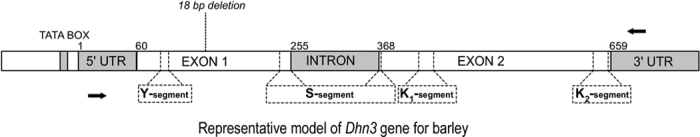
General structure of the *Dhn3* locus in *H. vulgare* (Choi *et al.* 1999) that is conserved in *H. spontaneum*. Arrows show the location of primers used to amplify *Dhn3* alleles in this study.

The *H. spontaneum Dhn3 (HsDhn3)* gene has typically 486 bp of coding sequences with two exons and 439 bp of noncoding sequences (59-bp 5′UTR, 113-bp intron and 267-bp 3′UTR) like *Hordeum vulgare Dhn3* (Fig. 1). We sequenced a 692-bp region of the *Dhn3* alelle including all the coding regions (195-bp Exon1 and 291-bp Exon 2) and 206 bp of noncoding regions. Through all sequences, we detected total 29 SNPs in the coding regions and intron of *HsDhn3*. The variation in the intron, with one SNP every 14 bp on average, was one-and-a-half fold as high as in the coding regions, with one SNP every 23 bp on average. There was only one gap observed in the sequenced region of the *HsDhn3*, 18-bp length in Exon 1. This gap was observed in only three *H. spontaneum* genotypes. The nucleotide diversity for the whole *HsDhn3* was estimated by Nei’s^37^ π statistics to be 0.00684 for the whole sequence. There was higher diversity in the intron region (π = 0.01247) than in the exon regions (π = 0.00290 and 0.00720 for Exon 1 and Exon 2, respectively). 16 haplotypes were observed for all regions of *Dhn3*, where the highest haplotype score was observed in Exon 2 with 12 haplotypes. The allelic variation was measured according to haplotype diversity (Hd; Table 1). The lowest value was found in the Exon 1 (Hd: 0.458), while Exon 2 showed the highest variability (Hd: 0.824). The Hd value was smaller in the intron (0.795) than Exon 2.

The SNP number, nucleotide diversity, and haplotype diversity were also calculated for each sub-region of *HsDhn3* gene (Table 1). These sub-regions are highly conserved regions containing one Y-segment, one S-segment, and two K-segments. There was also a spacer, named K_sp_, in-between the two K-segments. 10 of the 21 SNPs were observed in the K_sp_ region. The K_sp_ region also showed the highest scores for the number of haplotypes and haplotype diversity. The variation in K_1_ and K_2_ was detected with one SNP every 10 and 9 bp, respectively. Nucleotide diversity of K_1_ (π = 0.01354) is higher than K_2_ (π = 0.01058). The lowest variation between regions was observed in Y with π = 0.00414.

The *HsDhn3* gene is GC rich with a content of 66.9%. In total, 21 SNP mutations were detected within the coding region of *HsDhn3* (Table 1). Regarding the nature of base mutations, transition mutations were 76.2% of total, while transversion mutations were about 23.8% (Table 2). A/G substitutions had the highest percentage at 57.1. While 6 SNPs were synonymous, 15 SNPs were non-synonymous and led to amino acid replacements.

### Biochemical features and motif structure of DHN3 in *H. spontaneum*

**Figure 2:**
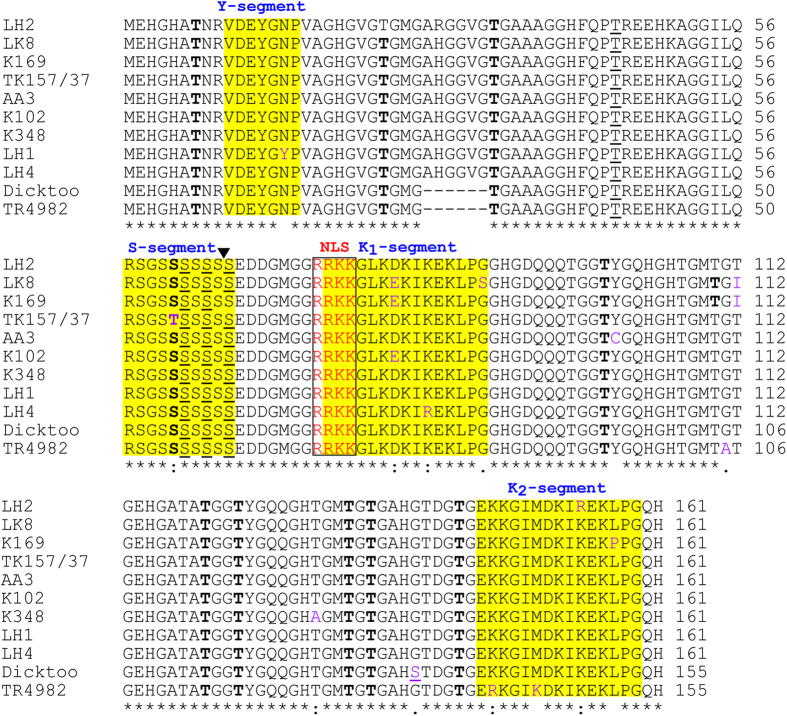
Multiple sequence alignment of the deduced amino acid sequences of the DHN3 proteins from *H. spontaneum* (9 haplotypes) and two *H. vulgare* genotypes (cvs TK157/37 and Dicktoo). The conserved segments (Y-, S-, and K-segments) are shown in yellow shade. The NLS segments are denoted in red and framed by a black line. The PKC phosphorylation sites are in boldface letters and the CK2 sites are underlined. The SNPs are shown in purple. The intron position is indicated by an arrow. Asterisks (*) indicates fully conserved residues, while colons (:) and periods (.) indicate less conserved residues.

The molecular weight of *Hordeum spontantenum* DHN3 (*Hs*DHN3) varied from 15.72 kDa to 16.22 kDa with 155 or 161 amino acid residues, respectively (see Supplementary Table S2 online). *Hs*DHN3s had a number of putative protein kinase C (PKC) phosphorylation sites, varying from 9 to 11, which was similar to that of *H. vulgare* (see Supplementary Table S2 online). PKC sites were outside of the conserved motifs, except a serine residue within the S-segment (Fig. 2). Aliphatic index values of *Hs*DHN3 were predicted to range from 32.19 to 35.22 (See Supplementary Table S2 online), thus showing high protein thermostability^48^. The instability index showed that the *Hs*DHN3 proteins were highly stable with values much lower than 40^40^. All *Hs*DHN3 were found to be highly hydrophilic, with GRAVY values ranging from −1.020 to −1.128 and also basic, with theoretical pIs varying from 7.99 to 8.90.

Similar to the DHN3 protein of cultivated barley, *Hs*DHN3 is YSK_2_-type containing one Y-segment, one S-segment and two K-segments (Fig. 2). The Y-segment sequences (DEYGNPV) were the same as in cultivated barley^14^, with the exception of the LH1 variant, which contains DEYG**Y**PV, where the amino acid Asn was replaced by Tyr. S-segments are Ser rich conserved motifs and typically described as RSGSSSSSSS^14^ and interrupted by an intron (Fig. 1). Although S-segments appear to be conserved in all *H. spontaneum* genotypes, Ser was replaced by Thr in *H. vulgare* cv. TK157/37 (Fig. 2).

In barley, the K segment has two 15-mer Lys-rich consensus segments RKKGLKDKIKEKLPG and EKKGIMDKIKEKLPG named the K_1_-segment and the K_2_-segment, respectively^14^. In the K_1_-segment, the amino acid Asp is replaced by Glu in the K102, K169, K394, and LK8 variants (Fig. 2). In addition, the LK8 variant included an amino acid change of Gly to Ser. In the K_1_ segment, another non-synonymous substitution included Lys replaced with Arg in the LH4 variant. Regarding the Φ-segments, there were conserved GHFQ, GDQQ, YGQH, and YGQQ sequences found between the Y-S and K_1_-K_2_ segments, similar to cv. Dicktoo in all *Hs*DHN3 variants with the exception of a Cys substitution occurring at position 101 in the Φ-segment of the AA3 variant. In addition, four amino acid substitutions, Gly to Ala, Thr to Ile, Thr to Ala, and Gly to Ser, were also observed between the K_1_- and K_2_- segments at positions 111, 112, 130, and 139, respectively.

**Figure 3:**
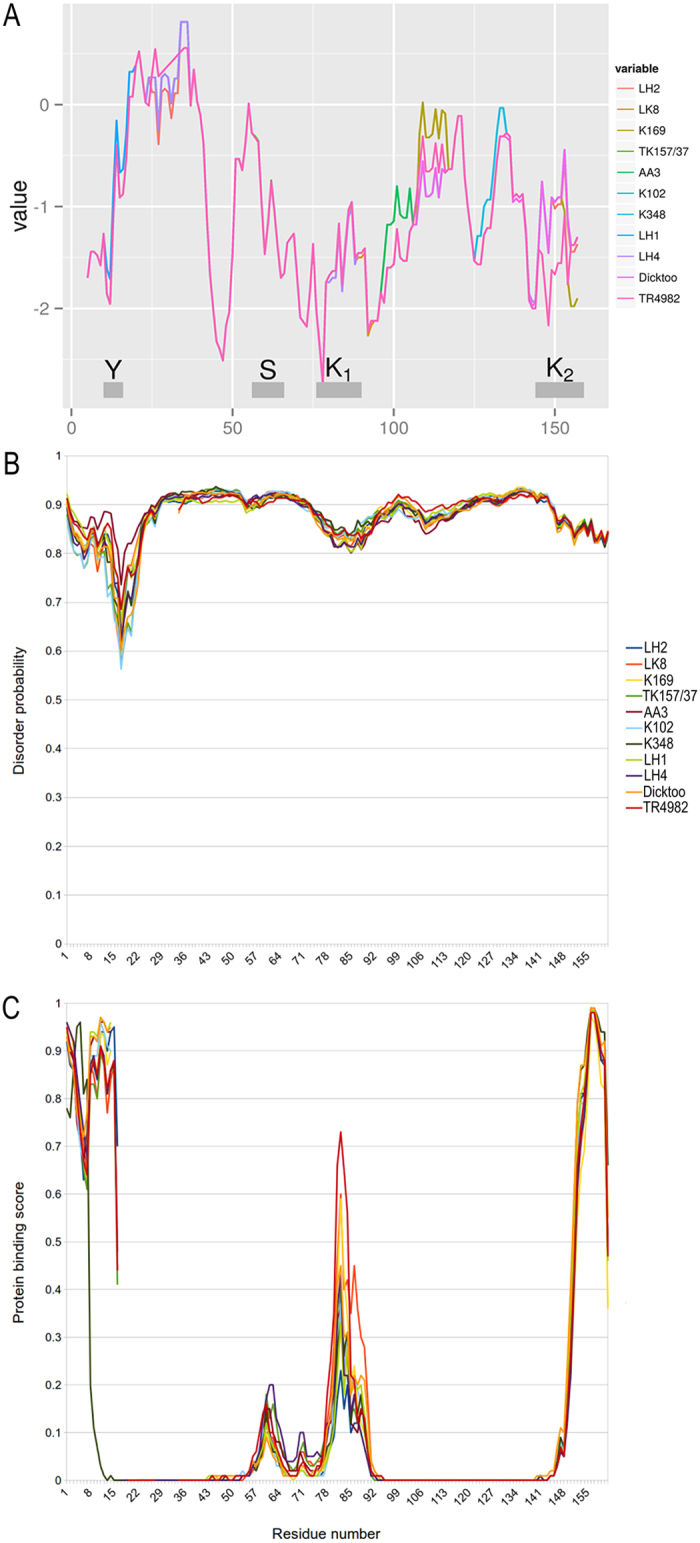
Structural characteristics of DHN3 in *H. spontaneum* genotyes and *H. vulgare* cvs. TK157/37 and Dicktoo. (**A**) Hydrophobicity values according to Kyle-Doolittle (1982). (**B**) Disorder probability predicted by DISOclust via the IntFOLD server. (**C**) Probability of protein binding amino acids predicted using DISOPRED3.

*Hs*DHN3 is hydrophilic due to the presence of K-segments (Fig. 3A). Additionally, the region between position 40 and 150 was found to be both hydrophilic and disordered in all *Hs*DHN3 (Fig. 3B). At position 145 we observed that TR4982 replaces a Lys by an Arg that results in an increased hydophilicity (Fig. 3A).

### Comparison of DHN3 sequences in cereal species

We compared the predicted DHN3 proteins from *H. spontaneum*, versus other closely related cereals in terms of their general biochemical properties (Table 3). All DHN3 proteins were YSK_2_-type, with the exception of *T. urartu* DHN3 (YSK-type). The number of amino acids varied from 154 (*S. bicolor*) to 183 residues (*B. distachyon*), while molecular weights ranged between 15.73 kDa (*A. tauschii*) and 18.93 kDa (*T. urartu*). All DHN3 proteins were stable, with an instability index (II) under 40, with the exception *T. urartu* (41.12). The most basic protein among the DHN3s was *T. urartu* DHN3 with a pI of 10.22. The number of predicted phosphorylation sites varied from 4 to 15 for PKC and 1 to 5 for CK2 in DHN3s (Table 3). All the DHN3 proteins were identified to be highly hydrophilic with GRAVY values ranging from −0.946 to −1.145. *H. spontaneum* variants contain on average 55.5% of charged and polar amino acids. The most frequent amino acid is Gly, a non-polar one, which constitutes 26.7% of the amino acid content. The frequency of the Cys and Phe are less than 1%. Cys was discovered only in the *H. spontaneum* variant AA3 and is a rare amino acid in *Dhn* genes (Fig. 2). Trp residues were not detected among any of the DHN3 proteins (see Supplementary Table S3 online).

**Figure 4:**
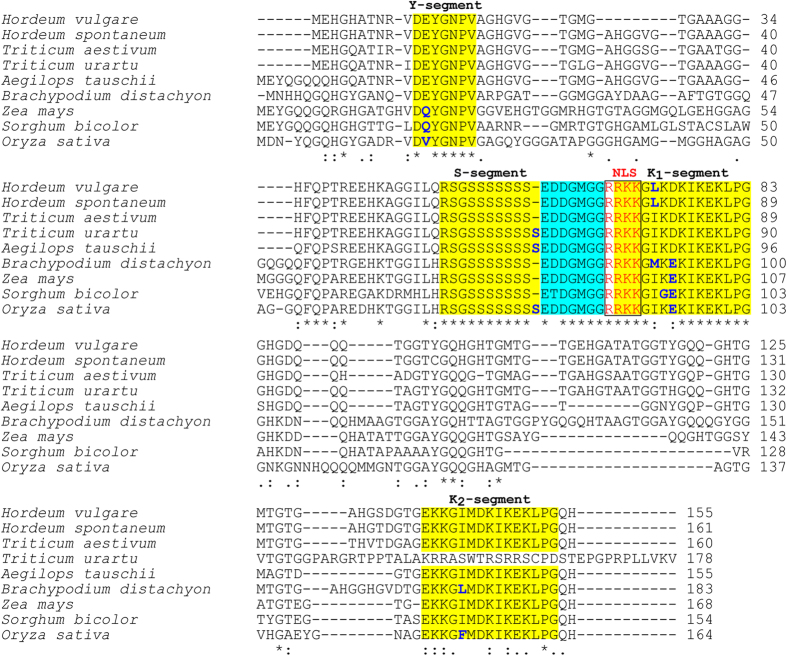
Multiple alignments of the predicted amino acid sequences of the DHN3 protein from *H. spontaneum* (consensus sequence), along with the ortholog proteins in other cereal species. The conserved segments are shown in yellow shade. The NLS segments are denoted in red and framed by a black line. A novel conserved sequence is shown in blue shade. Amino acid substitutions in conserved regions (Y-, S- , and K-segments) are denoted in blue. Asterisks (*) indicates fully conserved residues, while colons (:) and periods (.) indicate less conserved residues.

Comparisons of the predicted DHN3 proteins in different cereal species indicate that the Y-, S-, and K-segments are highly conserved sequences (Fig. 4). A consensus motif of [V/T]D[E/Q]YGNP (the Y-segment), located near the N-terminus was found in all cereal DHN3s. The Val, the first amino acid of the Y segment, was replaced by Ile and Leu in *T. urartu* and *S. bicolor*, respectively. In addition, the E/Q to V substitution is also present in *O. sativa* DHN3. The S-segment (RSGSSSSSS) was conserved intact with an extra Ser in *T. urartu*, *A. tauschii* and *O. sativa*.

The NLS peptide (RRKK), placed just upstream from the K_1_ segment (first K-segment), was found in all cereal DHN3s (Fig. 4). Although, the K_1_ segment (RKKGIKDKIKEKLPG) was found highly conserved in all cereals, some amino acid substitutions were discovered in the K_1_ segment. A non-polar amino acid Ile was replaced with Leu and Met, which were also non-polar. The positively charged Lys was substituted with the non-charged Gly in the *S. bicolor* DHN3 protein. In addition, there was another amino acid replacement between Asp and Glu in *B. distachyon*, *Z. mays*, *S. bicolor* and *O. sativa*. Although DHN3s have two highly conserved and Lys-rich segments named K_1_ and K_2_ in all cereals, the K_2_-segment occurring at the C-terminus (EKKGIMDKIKEKLPG) was not detected in the *T. urartu* DHN3. Two substitutions were discovered in the K_2_ segments at the same amino acid position. An Ile residue was replaced by Leu and Phe in *B. distachyon* and *O. sativa*, respectively. Another highly conserved region, [E(D/T)DGMGGR], not previously reported, was discovered between the S-segment and the K_1_-segment in all cereal DHN3s (Fig. 4). Only a single amino acid replacement, Asp to Thr, was found in the *S. bicolor* DHN3 for this conserved sub-sequence.

### Structural predictions for DHN3 variants

As expected, the 3D models predicted by IntFOLD server strongly suggest that all *Hs*DHN3 variants are mostly unstructured. No high quality globular 3D models were obtained; all models were highly variable and most of the models obtained were neither folded nor compact. The DISOclust results from the IntFOLD server, shown in Fig. 3B, also confirmed the extent of the intrinsic disorder for each of the variants, due to the large variations in the 3D locations of residues across the multiple alternative 3D models. The results in Fig. 3C and Supplemantary Fig. S1 indicate the putative regions of protein binding to be in the first 10–15 residues and the last 10–15 residues with a peak in the region around residue ~80. Often intrinsically disordered regions in proteins coincide with protein binding sites and the latest version of the DISOPRED method provides confidence scores for protein binding residues. Differences in protein binding profiles were observed between e.g. cultivated barley (TK157/37) and TR4982 with varying peak sizes occurring in the K1-segment around amino acid position ~80 (see Supplemantary Fig. S1). The varying confidence scores indicate that the SNPs may affect the putative protein binding function of *Hs*DHN3. Interestingly, the protein binding regions coincide with point mutations in the KxKIxEKLPx subsequence and in the C-terminal of K-segment (Fig. 3C).

## Discussion

Dehydrins play a fundamental role in the response of plants to different abiotic stresses especially dehydration, salinity and low temperatures by accumulating in vegetative tissues. They are the best-investigated group within LEA proteins with the characterized multilocus families including ten members in *Arabidopsis*^13^, eight members in rice^49^, fifty-four unigene in wheat^50^, and thirteen members in barley^14^,^15^. Dehydrins are characterized by the presence and copy number of several conserved motifs named the K-, S-, and Y-segments. DHN3 from wild barley is YSK_2_-type and structurally highly similar to cultivated barley. Interestingly, a 18-bp deletion occurring on Exon 1 was determined in only three out of 21 *H. spontaneum* genotypes: TR4982 (Çanakkale), TR47002 (İzmir) and TR49085 (Adıyaman). Different polypeptide size as a result of the indel was previously reported in *H. vulgare* cv. Himalaya and cv. Dicktoo^14^. Dehydrins are known to be located in different compartments of cell, including the cytoplasm, nucleus, mitochondria, chloroplast, and vicinity of plasma membrane^31^. YSK_2_-type dehydrins have both cytoplasmic and nuclear localizations but are mostly found in the nucleus^51^,^52^. Goday *et al.*^51^ also reported the *in vitro* interaction of a maize DHN5 homolog, RAB17 with a SV40 NLS signal for its import to nucleus. In our study, a “RRKK” motif, postulated as a nuclear localization signal (NLS), was determined just upstream from the first K-segment of nine cereal DHN3s (Fig. 4). Further experimental data is needed to confirm the functionality of the NLS sequence as well as the exact localization of DHN3s in barley.

In general, cereal DHN3s were found to be stable proteins except the *T. urartu* (with an instability index of 41.12). The presence of only one K-segment in *T. urartu* DHN3 (*Tu*DHN3), in contrast to other dehydrins, may have a negative effect on protein stability. On the other hand, *Tu*DHN3 was also the most basic protein with the highest molecular weight (18.93 kDa). *T. urartu* is a wild diploid wheat and progenitor species of a genome of bread wheat. Despite the sparse *T. urartu* literature, LEA proteins have been recently found associated with cold tolerance in this species^53^. The NetphosK 1.0 program predicted that *Hs*DHN3 might be specifically phosphorylated by protein kinase C at 9-11 sites, which were mainly Thr residues. Y_n_SK_n_-type DHNs are predominantly phosphorylated by protein kinase C group proteins, rather than CK2s^29^. Compared to other cereal species, *H. vulgare* and *H. spontaneum* had one of the highest occurrences of PKC phosphorylation sites, second only to *T. urartu.* Particularly, phosphorylation by protein kinase C at K-segments has been found to be associated with membrane binding functions of dehydrins^29^. Therefore, both *Hs*DHN3 and *Tu*DHN3 are good candidates to investigate membrane-dehydrin interactions. Amino acid changes led to the occurrence of a new phosphorylation site in two *H. spontaneum* accessions, LK8 and K169, by replacement of Thr at the position of 112. Brini *et al.*^52^ found that the phosphorylation pattern in wheat DHNs was related to abiotic stress tolerance. In particular, higher phosphorylation indicated higher tolerance to drought and salinity. This suggests that the extra phosphorylation site may play a role in the drought tolerance of LK8 and K169.

The amino acid composition of *Hs*DHN3 showed a high proportion of Gly residues (26.7%) conferring flexibility to the protein with the lack of a hydrophobic core and other factors. Moreover, 55.5% of *Hs*DHN3 amino acids were polar amino acids with hydrophilic character and this was also supported by the GRAVY results, with calculated values ranging from −1.020 to −1.128. In general, DHN3s are Gly-rich proteins and known to be deficient in Trp and Cys in the literature. We have found a Cys residue in the *H. spontaneum* variant AA3. *S. bicolar* and *T. urartu* contained one Cys (0.6%) and one Trp (0.6%) residue among the cereal DHN3s. Intrinsically disordered proteins are also significantly depleted in Cys and Trp^54^; typically less than 1%, compared with the average folded protein in the Protein Databank. In general, His residues are rarely found in proteins and constitute approximately 2% of the amino acid content^55^,^56^. Nevertheless, dehydrins contain a higher proportion of His residues. For example, His content ranged from 3.2% to 13.5% in *Arabidopsis* DHNs^56^. We have found that *Hs*DHN3s were relatively His rich proteins containing 8.1% His residues. Moreover, conserved His residues were adjacent, with both K-segments formed as Gly-His (GH) or Gln-His (QH) motifs in *Hs*DHN3. Eriksson *et al.*^29^ reported that the ionization state of His residues flanking the K-segments modulates the affinity of dehydrins to the cellular membranes in a pH dependent manner. His residues were not concentrated as motifs in cereal DHN3s, as earlier reported for a Citrus dehydrin^56^.

The *Hs*DHN3 protein variants are all likely to be intrinsically disordered (natively unstructured) with a likelihood of protein binding sites near the N- and C-termini and surrounding residue 80. The C-terminal site and the site around residue 80 also coincide with predictions of alpha helices and so these regions may undergo and disorder-order transition on protein binding. Importantly, the point mutations are observed occur within these protein binding regions and therefore the amino acid substitutions in these sequence variants may affect protein interactions. Similarly, the protein binding regions coincide with point mutations in the KxKIxEKLPx subsequence and in the C-terminal K-segment, a region highly conserved in plants^27^. Often disordered regions become ordered on binding, so it is interesting to predict secondary structures to determine if local structures may form during protein-protein interactions. The specific nature of these interactions is not yet known although the predicted helices are unlikely to form coiled-coil interactions according to results obtained from Pcoils. DHNs are also known to interact with lipids, membranes, metal ions, water, ice and DNA^31^. They function as cryoprotectant and have binding properties that allow chaperon activities. However, the exact mechanism and details of these interactions are not completely clear. Recently, dehydrin-dehydrin binding has been demonstrated in two plant species^57^,^58^. Yeast two-hybrid assays confirmed that K-segments and His residues are required for dimerization of *Opuntia* DHN1^58^. In our study, the peaks shown in Fig. 3C and Supplemantary Fig. S1 indicated disordered residues that may fold or become ordered upon protein binding. These regions are therefore likely to be the dimerization sites in the *Hs*DHN3 variants.

In this study, we report the genetic structure and diversity of near-complete *Dhn3* alleles from native *H. spontaneum* plants. By taking the advantage of the additional data, we were able to compare predicted DHN3 sequences with other closely related cereals, which allowed us to distinguish polymorphisms and motif structures. Most of the SNPs identified occurred in non-coding and inter-segment positions and resulted non-synonymous mutations. However, point mutations in several variants have resulted in amino acids with opposite chemical properties as seen in the substitution of Met (a sulphur containing hydrophobic) for Lys (a basic, polar, and positively charged), or Gly (Aliphatic and nonpolar) for Ser (Non-aromatic hydroxyl containing, polar). Dehydrins, as IDPs, are structurally not globular folded molecules; however they are proposed to be rich in functionality because of their flexibility and modularity. Bioinformatics tools such as, IntFOLD, DISOclust and DISOPRED are ideal for deducing the nature and the functional properties of plant IDPs, and act as a guide for further experimental studies. From the predictions in our work, we have showed the potential availability of at least one likely protein binding site in barley DHN3. Furthermore, point mutations within the conserved sequences in *H. spontaneum* variants affected the predicted protein binding profile. Our results may contribute to future experimental designs to resolve the interactions of barley DHNs with known and undetermined ligands, which lead to their diverse functions in plant cells as cryoprotectants and chaperons.

## Additional Information

**How to cite this article**: Uçarlı, C. *et al.* Genetic diversity at the *Dhn3* locus in Turkish *Hordeum spontaneum* populations with comparative structural analyses. *Sci. Rep.* **6**, 20966; doi: 10.1038/srep20966 (2016).

## Supplementary Material

Supplementary Information

## Figures and Tables

**Table 1:** Summary statistics of nucleotide and haplotype diversity in the coding region and conserved motifs of the *Dhn3* gene of *H. spontaneum*.

Region/Motif	Fragmentlength (bp)	No. ofSNP	Deletion	Haplotype	Haplotypediversity (Hd)	Nucloetidediversity (π)
Exon 1	195	5	+	6	0.458	0.00290
Exon 2	291	16	−	12	0.824	0.00720
Intron	113	8	−	8	0.795	0.01247
All	599	29	+	16	0.948	0.00684
Y	21	1	−	2	0.095	0.00454
S	30	1	−	2	0.095	0.00317
K_1_	45	4	−	5	0.486	0.01354
K_sp_	234	10	−	10	0.776	0.00651
K_2_	45	5	−	5	0.352	0.01058
All	375	21	−	12	0.824	0.00747

**Table 2:** Summary of nucleotide changes in the coding region of *H. spontaneum Dhn3* according to the consensus sequence.

	Substitutiontypes	No. of nucleotidesubstitution	% of nucleotidesubstitution	No. of amino acidsubstitution
***Transition***	A>G	8	57.1	7
	G>A	4		1
	C>T	2	19.1	2
	T>C	2		1
	Subtotal	16	76.2	11
***Transversion***	A>C	0	0	0
	C>A	0		0
	G>T	0	0	0
	T>G	0		0
	A>T	1	9.5	1
	T>A	1		1
	G>C	1	14.3	1
	C>G	2		1
	Subtotal	5	23.8	4
	**Total**	**21**	**100**	**15**

**Table 3:** Biochemical characteristics of DHN3 protein from *H. spontaneum* and the closely related cereals listed in Materials and Methods section.

Species	Access No.	Type	No. ofresidues	MW(kDa)	PKCNo	CK2No	pI	GRAVY	AI	II
*H. spontaneum*	Consensus	YSK_2_	155–161*	16.13	9–11	4	8.24	−1.051	33.07	13.26
*H. vulgare*	AF043089.1	YSK_2_	155	15.86	10	4	8.08	−1.076	32.18	12.42
*T. aestivum*	AAL50791	YSK_2_	160	15.97	8	4	8.06	−0.946	36.69	16.06
*T. urartu*	EMS45466.1	YSK	178	18.93	15	5	10.22	−0.946	40.11	41.12
*A. tauschii*	EMT24840	YSK_2_	155	15.73	8	5	8.83	−1.125	34.06	26.81
*B. distachyon*	XP003574997	YSK_2_	183	18.46	6	2	9.25	−1.101	27.87	18.26
*Z.mays*	CAM56274.1	YSK_2_	168	17.06	8	3	8.78	−1.145	31.43	25.27
*O. sativa*	NP001067843	YSK_2_	164	16.54	4	4	9.27	−1.033	32.26	24.32
*S. bicolar*	AAA19693	YSK_2_	154	16.28	6	1	9.67	−1.002	43.25	25.40

MW (Molecular Weight), PKC (Protein Kinase C), CK2 (Casein Kinase 2), pI (Isoelectric Point), GRAVY (Grand Average of Hydropathy), AI (Aliphatic Index), and II (Instability Index) were predicted based on amino acid composition. .* Two values due to the presence of 6-aa indel in Exon 1.

## References

[b1] Zohary D. & Hopf M. Domestication of plants in the Old World. The origin and spread of cultivated plants in West Asia, Europe and the Nile Valley. Clarendon Press, Oxford, England (1993).

[b2] Badr A. *et al.* On the origin and domestication history of barley (*Hordeum vulgare*). Mol. Biol. Evol. 17, 499–510 (2000). 10742042 10.1093/oxfordjournals.molbev.a026330

[b3] Knüpffer H., Terentyeva I., Hammer K., Kovelava O. & Sato K. Ecogeographical diversity – a Vavilovian approach in *Diversity in barley* (*Hordeum vulgare*), (eds von Bothmer R. *et al.*) 53–76 (Elsevier, 2003).

[b4] Forster B. P. *et al.* Locating genotypes and genes for abiotic stress tolerance in barley: a strategy using maps, markers and the wild species. New Phytologist. 137, 141–147 (1997).

[b5] Nevo E. & Chen G. Drought and salt tolerances in wild relatives for wheat and barley improvement. Plant Cell Environ. 33, 670–685 (2010) 20040064 10.1111/j.1365-3040.2009.02107.x

[b6] Dure L. & Galau G. A. Developmental biochemistry of cotton seed embryogenesis and germination. XIII. Regulation of biosynthesis of principal storage proteins. Plant Physiol. 68, 187–194 (1981). 16661868 10.1104/pp.68.1.187 PMC425913

[b7] Dure L. *et al.* (1989) Common amino acid sequence domains among the LEA proteins of higher plants. Plant Mol. Biol. 12, 475–486 (1989). 24271064 10.1007/BF00036962

[b8] Bartels D. & Sunkar R. Drought and salt tolerance in plants. CRC. Crit. Rev. Plant Sci. 24, 23–58 (2005).

[b9] Close T. J. Dehydrins: Emergence of a biochemical role of a family of plant dehydration proteins. Physiol. Plant. 97, 795–803 (1996).

[b10] Zhu B., Choi D. W., Fenton R. & Close T. J. Expression of the barley dehydrin multigene family and the development of freezing tolerance. Mol. Gen. Genet. 264, 145–153 (2000). 11016844 10.1007/s004380000299

[b11] Tommasini L. *et al.* Dehydrin gene expression provides an indicator of low temperature and drought stress: transcriptome-based analysis of barley (*Hordeum* vulgare L.). Funct. Integr. Genomics. 8, 387–405 (2008). 18512091 10.1007/s10142-008-0081-z

[b12] Kleinwächter M., Radwan A., Hara M. & Selmar D. Dehydrin expression in seeds: an issue of maturation drying. Front. Plant. Sci. 5, 402 (2014). 25221559 10.3389/fpls.2014.00402 PMC4145252

[b13] Hundertmark M. & Hincha D. K. LEA (late embryogenesis abundant) proteins and their encoding genes in *Arabidopsis thaliana*. BMC Genomics. 9, 118 (2008). 18318901 10.1186/1471-2164-9-118 PMC2292704

[b14] Choi D. W., Close T. J. & Zhu B. The barley (*Hordeum vulgare* L.) dehydrin multigene family: sequences, allele types, chromosome assignments, and expression characteristics of 11 Dhn genes of cv. Dicktoo. Theor. Appl. Genet. 98, 1234–1247 (1999).

[b15] Rodriguez E. M., Svensson J. T., Malatrasi M., Choi D. W. & Close T. J. Barley Dhn13 encodes a KS-type dehydrin with constitutive and stress responsive expression. Theor. Appl. Genet. 110, 852–858 (2005). 15711789 10.1007/s00122-004-1877-4

[b16] Close T. J., Fenton R. D. & Moonann F. A view of plant dehydrins using antibodies specific to the carboxy terminal peptide. Plant Mol. Biol. 23, 279–286 (1993). 7693020 10.1007/BF00029004

[b17] Close T. J. Dehydrins: a commonalty in the response of plants to de- hydration and low temperature. Physiol. Plant. 100, 291–296 (1997).

[b18] Park S. Y. *et al.* Rapid upregulation of *Dehyrin3* and *Dehydrin4* in response to dehydration is a characteristic of drought-tolerant genotypes in barley. J. Plant Biol. 49, 455–462 (2006)

[b19] Guo P. *et al.* Differentially expressed genes between drought-tolerant and drought-sensitive barley genotypes in response to drought stress during the reproductive stage. J. Exp. Bot. 60, 3531–3544 (2009). 19561048 10.1093/jxb/erp194 PMC2724701

[b20] Du J. B. *et al.* Comparative expression analysis of dehydrins between two barley varieties, wild barley and Tibetan hulless barley associated with different stress resistance. Acta Physiol. Plant. 33, 567–57 (2011).

[b21] Karami A. *et al.* Expression analysis of dehydrin multigene family across tolerant and susceptible barley (*Hordeum vulgare* L.) genotypes in response to terminal drought stress. Acta Physiol. Plant. 35, 2289–2297 (2013).

[b22] Suprunova T. *et al.* Differential expression of dehydrin genes in wild barley, *Hordeum spontaneum*, associated with resistance to water deficit. Plant, Cell Environ. 27, 1297–1308 (2004)

[b23] Hughes S. & Graether S. P. Cryoprotective mechanism of a small intrinsically disordered dehydrin protein. Protein Sci. 20, 42–50 (2011). 21031484 10.1002/pro.534 PMC3047060

[b24] Hughes S. L. *et al.* The importance of size and disorder in the cryoprotective effects of dehydrins. Plant Physiol. 163, 1376–1386 (2013). 24047864 10.1104/pp.113.226803 PMC3813657

[b25] Hara M., Terashima S. & Kuboi T. Characterization and cryo-protective activity of cold-responsive dehydrin from *Citrus unshiu*. J. Plant Physiol. 158, 1333–1339 (2001).

[b26] Alsheikh M. K., Heyen B. J. & Randall S. K. (2003) Ion binding properties of the dehydrin ERD14 are dependent upon phosphorylation. J. Biol. Chem. 278, 40882–40889 (2003). 12917402 10.1074/jbc.M307151200

[b27] Koag M. C. *et al.* The K-segment of maize DHN1 mediates binding to anionic phospholipid vesicles and concomitant structural changes. Plant Physiol. 150, 1503–1514 (2009). 19439573 10.1104/pp.109.136697 PMC2705017

[b28] Tompa P. *et al.* Protein-water and protein-buffer interactions in the aqueous solution of an intrinsically unstructured plant dehydrin: NMR intensity and DSC aspects. Biophys. J. 91, 2243–2249 (2006). 16798808 10.1529/biophysj.106.084723 PMC1557563

[b29] Eriksson S. K., Kutzer M., Procek J., Gröbner G. & Harryson P. Tunable membrane binding of the intrinsically disordered dehydrin Lti30, a cold-induced plant stress protein. Plant Cell 23, 2391–2404 (2011). 21665998 10.1105/tpc.111.085183 PMC3160030

[b30] Drira M. *et al.* The K-segments of the wheat dehydrin DHN-5 are essential for the protection of lactate dehydrogenase and β-glucosidase activities *in vitro*. Mol. Biotechnol. 54, 643–650 (2013). 23054631 10.1007/s12033-012-9606-8

[b31] Graether S. P. & Boddington K. F. Disorder and function: a review of the dehydrin protein family. Front. Plant Sci. 5, 576 (2014). 25400646 10.3389/fpls.2014.00576 PMC4215689

[b32] Atkins J. D., Boateng S. Y., Sorensen T. & McGuffin L. J. Disorder prediction methods, their applicability to different protein targets and their usefulness for guiding experimental studies. Int. J. Mol. Sci. 16, 19040–19054 (2015). 26287166 10.3390/ijms160819040 PMC4581285

[b33] Weining S. & Langridge P. Identification and mapping of polymorphisms in cereals based on polymerase chain reaction. Theor. Appl. Genet. 82, 209–216 (1991). 24213068 10.1007/BF00226215

[b34] Altschul S. F., Gish W., Miller W., Myers E. W. & Lipman D. J. Basic local alignment search tool. J. Mol. Biol. 215, 403–410 (1990). 2231712 10.1016/S0022-2836(05)80360-2

[b35] Larkin M. A. *et al.* Clustal W and Clustal X version 2.0. Bioinformatics. 23, 2947–8 (2007). 17846036 10.1093/bioinformatics/btm404

[b36] Tamura K., Stecher G., Peterson D., Filipski A. & Kumar S. MEGA6: Molecular Evolutionary Genetics Analysis version 6.0. Mol. Biol. Evol. 30, 2725–9 (2013). 24132122 10.1093/molbev/mst197 PMC3840312

[b37] Nei M. In Molecular evolutionary genetics. (Columbia University Press, 1987).

[b38] Librado P. & Rozas J. (2009) DnaSP v5: a software for comprehensive analysis of DNA polymorphism data. Bioinformatics. 25, 1451–2 (2009). 19346325 10.1093/bioinformatics/btp187

[b39] Kyte J. & Doolittle R. A simple method for displaying the hydropathic character of a protein. J. Mol. Biol. 157, 105–132 (1982). 7108955 10.1016/0022-2836(82)90515-0

[b40] Gasteiger E. *et al.* Protein Identification and Analysis Tools on the ExPASy Server in The proteomics protocols handbook (ed. Walker J. M.) 571–607 (Humana, 2005).

[b41] Blom N., Sicheritz-Ponten T., Gupta R., Gammeltoft S. & Brunak S. Prediction of post-translational glycosylation and phosphorylation of proteins from the amino acid sequence. Proteomics. 4, 1633–1649 (2004). 15174133 10.1002/pmic.200300771

[b42] Roche D. B., Buenavista M. T., Tetchner S. J. & McGuffin L. J. The IntFOLD server: an integrated web resource for protein fold recognition, 3D model quality assessment, intrinsic disorder prediction, domain prediction and ligand binding site prediction. Nucleic Acids Res. 39, 171–176 (2011). 10.1093/nar/gkr184 PMC3125722 21459847

[b43] McGuffin L. J., Atkins J., Salehe B. R., Shuid A. N. & Roche D. B. IntFOLD: an integrated server for modelling protein structures and functions from amino acid sequences. Nucleic Acids Res. 43, W169–73 (2015). 25820431 10.1093/nar/gkv236 PMC4489238

[b44] Buenavista M. T., Roche D. B. & McGuffin L. J. Improvement of 3D protein models using multiple templates guided by single-template model quality assessment. Bioinformatics. 28, 1851–1857 (2012). 22592378 10.1093/bioinformatics/bts292

[b45] McGuffin L. J. Intrinsic disorder prediction from the analysis of multiple protein fold recognition models. Bioinformatics. 24, 1798–1804 (2008). 18579567 10.1093/bioinformatics/btn326

[b46] Ward J. J. *et al.* Prediction and functional analysis of native disorder in proteins from the three kingdoms of life. J. Mol. Biol. 337, 635–645(2004). 15019783 10.1016/j.jmb.2004.02.002

[b47] Jones D. T. & Cozzetto D. DISOPRED3: Precise disordered region predictions with annotated protein binding activity. Bioinformatics. 31, 857–863 (2015). 25391399 10.1093/bioinformatics/btu744 PMC4380029

[b48] Ikai A. J. Thermostability and aliphatic index of globular proteins. J. Biochem. 88, 1895–1898 (1980). 7462208

[b49] Wang X. S. *et al.* Genome-scale identification and analysis of LEA genes in rice (*Oryza sativa* L.). Plant Sci. 172, 414–420 (2007).

[b50] Wang Y. *et al.* Classification and expression diversification of wheat dehydrin genes. Plant Sci. 214, 113–120 (2014). 24268169 10.1016/j.plantsci.2013.10.005

[b51] Goday A. *et al.* The maize abscisic acid-responsive protein RAB17 is located in the nucleus and interacts with nuclearlocalization signals. Plant Cell. 6, 351–360 (1994). 8180497 10.1105/tpc.6.3.351 PMC160438

[b52] Brini F. *et al.* Functional characterisation of DHN-5, a dehydrin showing a differential phosphorylation pattern in two Tunisian durum wheat (*Triticum durum* Desf.) varieties with marked differences in salt and drought tolerance. Plant Sci. 172, 20–28 (2007).

[b53] Gharechahi J., Alizadeh H., Naghavi M. R. & Sharifi G. A proteomic analysis to identify cold acclimation associated proteins in wild wheat (*Triticum urartu* L.). Mol Biol Rep. 41, 3897–3905 (2014). 24535272 10.1007/s11033-014-3257-8

[b54] Tompa P. Intrisically unstructured proteins. Trends Biochem. Sci. 27, 527–533 (2002). 12368089 10.1016/s0968-0004(02)02169-2

[b55] Ueda E. K. M., Gout P. W. & Morganti L. Current and prospective applications of metal ion-protein binding. J. Chromatogr. A. 988, 1–23 (2003). 12647817 10.1016/s0021-9673(02)02057-5

[b56] Hara M., Fujinaga M. & Kuboi T. Metal binding by citrus dehydrin with histidine-rich domains. J. Exp. Bot. 56, 2695–2703 (2005). 16131509 10.1093/jxb/eri262

[b57] Rahman L. N. *et al.* Interactions of *Thellungiella salsuginea* dehydrins TsDHN-1 and TsDHN-2 with membranes at cold and ambient temperatures-surface morphology and single-molecule force measurements show phase separation, and reveal tertiary and quaternary associations. Biochim. Biophys. Acta. 1828, 967–980 (2013). 23219803 10.1016/j.bbamem.2012.11.031

[b58] Hernández-Sánchez I. E. *et al.* Nuclear localization of the dehydrin OpsDHN1 is determined by histidine-rich motif. Front. Plant Sci. 6, 1–8 (2015). 26442018 10.3389/fpls.2015.00702 PMC4561349

