# Supplementary material for: Genetic diversity at the Dhn3 locus in Turkish Hordeum spontaneum populations with comparative structural analyses
Source: Sci Rep. 2016 Feb 12;6:20966. doi: 10.1038/srep20966 (PMC4751488; doi:10.1038/srep20966)

Title:

Genetic diversity at the *Dhn3* locus in Turkish *Hordeum spontaneum* populations with comparative structural analyses

Authors

Cüneyt Uçarlı[1], Liam J. McGuffin[2], Süleyman Çaputlu[1], Andres Aravena [1] and Filiz Gürel[1]*

[1] Department of Molecular Biology and Genetics, Istanbul University, Vezneciler 34134, Istanbul, Turkey

[2] School of Biological Sciences, University of Reading, Whiteknights, Reading RG6 6AS, UK

Supplementary Table S1. *Hordeum spontaneum* accessions used in the study.

Accession Code	Provenance	Seed provider
TR40812	Gaziantep	AARI
TR4982	Çanakkale	AARI
TR31623	Mardin	AARI
TR47002	İzmir	AARI
TR49085	Adıyaman	AARI
TR50358	Diyarbakır	AARI
TRHs01	Diyarbakır	Sabancı University
AA1	Diyarbakır	Dicle University
AA2	Diyarbakır	Dicle University
AA3	Diyarbakır	Dicle University
K1239	Diyarbakır (Karacadag)	DPPRI
K102	Diyarbakır (Karacadag)	DPPRI
K394	Diyarbakır (Karacadag)	DPPRI
K169	Diyarbakır (Karacadag)	DPPRI
K348	Diyarbakır (Karacadag)	DPPRI
LH1	Diyarbakır (near Hasankeyf)	Istanbul University
LH2	Diyarbakır (near Hasankeyf)	Istanbul University
LH4	Diyarbakır (near Hasankeyf)	Istanbul University
LH6	Diyarbakır (near Hasankeyf)	Istanbul University
LK8	Diyarbakır (Kapuzlu village)	Istanbul University
LK9	Diyarbakır (Kapuzlu village)	Istanbul University

AARI: Aegean Agricultural Research Institute; DPPRI: Diyarbakır Plant Protection Research Institute

Supplementary Table S2. Biochemical characteristics of nine haplotypes of *Hs*DHN3 proteins in *H. spontaneum*.

Accession	Type	No. of residues	MW (kDa)	PKC No	CK2 No	pI	GRAVY	AI	II
LK8	YSK_2	161	16.22	11	4	8.08	-1.020	35.22	13.70
K169	YSK_2	161	16.17	11	4	8.08	-1.051	32.80	13.33
AA3	YSK_2	161	16.10	10	4	7.99	-1.026	32.80	12.74
K102	YSK_2	161	16.17	10	4	8.08	-1.050	32.80	12.74
TR4982	YSK_2	155	15.72	10	4	8.87	-1.128	32.19	14.36
K348	YSK_2	161	16.13	10	4	8.08	-1.034	33.42	13.21
LH1	YSK_2	161	16.21	10	4	8.07	-1.036	32.80	13.16
LH2	YSK_2	161	16.21	9	4	8.90	-1.061	32.80	13.34
LH4	YSK_2	161	16.19	10	4	8.10	-1.053	32.80	12.74

MW (Molecular Weight), PKC (Protein Kinase C), CK2 (Casein Kinase 2), pI (Isoelectric Point), GRAVY (Grand Average of Hydropathy), AI (Aliphatic Index), and II (Instability Index) were predicted based on amino acid composition.

Supplementary Table S3 Relative frequency of predicted amino acids in DHN3 from *H. spontaneum* (mean values of 21 genotypes) and the closely related cereals listed in the Materials and Methods section. Values are percentages.

**Amino acid**	***H. spontaneum***	***H. vulgare***	***T. aestivum***	***T. urartu***	***A. tauschii***	***B. distachyon***	***Z. mays***	***O. sativa***	***S. bicolor***
Ala(A)	6.2	6.2	7.5	8.4	6.5	8.2	5.4	8.5	9.7
Arg(R)	3.1	3.1	3.1	7.3	3.2	2.7	3.6	3.0	5.8
Asn(N)	1.2	1.2	0.6	1.1	1.9	2.2	0.6	4.3	1.9
Asp(D)	4.3	4.3	5.0	3.4	4.5	3.8	3.6	4.3	3.2
Cys(C)	0.03	0.0	0.0	0.6	0.0	0.0	0.0	0.0	0.6
Gln(Q)	5.6	5.6	5.6	5.6	9.7	9.3	7.7	7.3	6.5
Glu(E)	5.8	5.6	5.0	3.9	5.2	3.8	6.5	4.3	5.8
Gly(G)	26.7	26.7	25.6	22.5	24.5	26.8	27.4	26.8	17.5
His(H)	8.1	8.1	6.9	5.6	5.2	6.6	7.1	5.5	6.5
Ile(I)	2.5	2.5	3.8	2.2	3.2	1.6	3.0	2.4	2.6
Leu(L)	2.5	2.5	1.9	3.4	1.9	2.2	2.4	1.8	4.5
Lys(K)	7.5	7.5	7.5	5.1	7.7	7.1	7.7	7.9	7.8
Met(M)	3.7	3.7	3.8	1.7	3.2	3.8	3.6	4.9	3.9
Phe(F)	0.6	0.6	0.6	0.6	0.6	1.1	0.6	1.2	0.6
Pro(P)	2.5	2.5	3.1	5.6	3.2	3.3	2.4	3.0	3.2
Ser(S)	5.0	5.0	6.2	7.3	7.1	4.4	6.0	5.5	7.1
Thr(T)	10.6	10.6	9.4	10.7	7.1	7.7	7.7	3.0	7.1
Trp(W)	0.0	0.0	0.0	0.6	0.0	0.0	0.0	0.0	0.6
Tyr(Y)	1.9	1.9	1.9	1.1	2.6	3.8	3.0	3.7	2.6
Val(V)	2.4	2.5	2.5	3.4	2.6	1.6	1.8	2.4	1.9

Supplementary Fig. S1 DISOPRED3 plots showing an alternative measure of disorder probability and the likely protein binding sites for each of the *Hs*DHN3 variants and barley. (A) TR4982 (B) TR49085 (C) K169 (D) LK8 (E) LH4 (F) TK157/37.

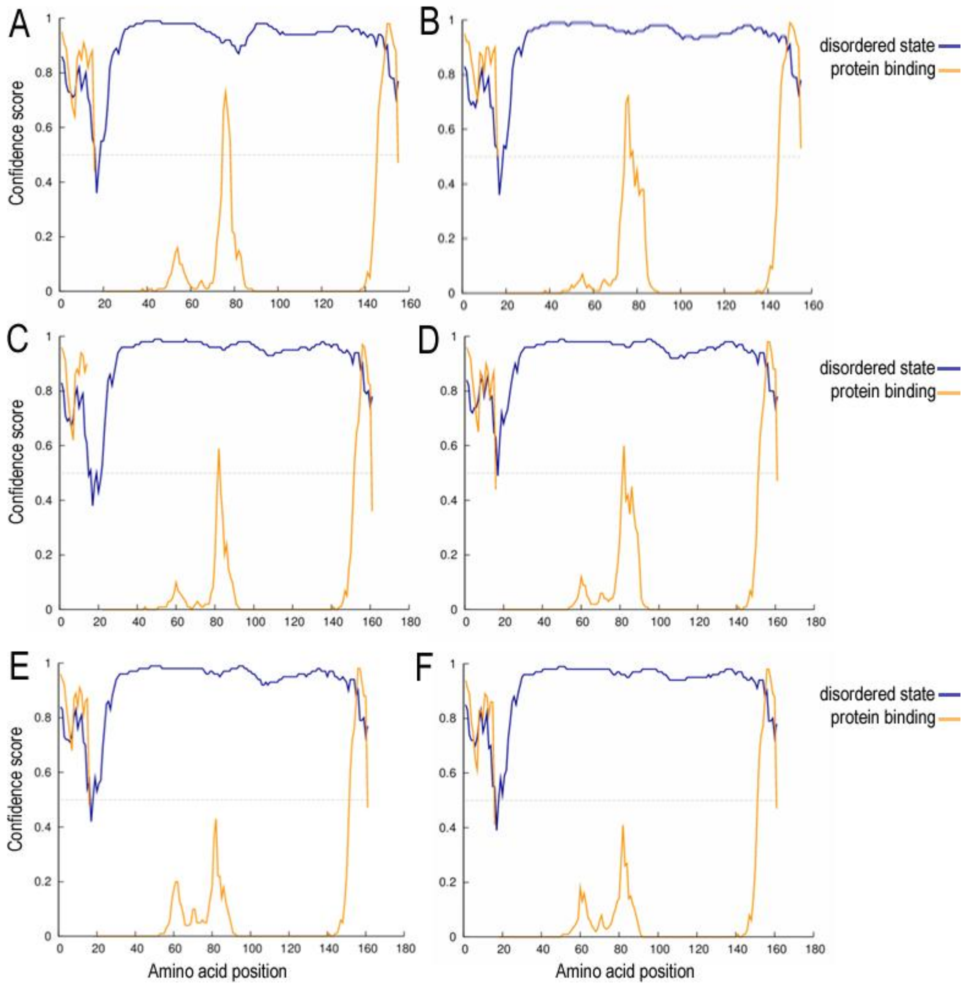

Supplement: Supplementary Information [file srep20966-s1.pdf]
